# Association of maternal thyroid peroxidase antibody during pregnancy with placental morphology and inflammatory and oxidative stress responses

**DOI:** 10.3389/fendo.2023.1182049

**Published:** 2023-09-22

**Authors:** Xue Ru, Mengting Yang, Yuzhu Teng, Yan Han, Yabin Hu, Jianqing Wang, Fangbiao Tao, Kun Huang

**Affiliations:** ^1^ Department of Maternal, Child & Adolescent Health, School of Public Health, Key Laboratory of Population Health Across Life Cycle, Anhui Medical University (AHMU), Ministry of Education of the People's Republic of China, National Health Commission Key Laboratory of Study on Abnormal Gametes and Reproductive Tract, Anhui Provincial Key Laboratory of Population Health and Aristogenics, Hefei, China; ^2^ Scientific Research Center in Preventive Medicine, School of Public Health, Anhui Medical University (AHMU), Hefei, China

**Keywords:** TPOAb, placental morphology, inflammation, oxidative stress, pregnancy, cytokines, cohort

## Abstract

**Background:**

Studies suggest that thyroid peroxidase antibody (TPOAb) positivity exposure during pregnancy may contribute to changes in placental morphology and pathophysiology. However, little is known about the association of maternal TPOAb during pregnancy with placental morphology and cytokines. This study focuses on the effect of repeated measurements of maternal TPOAb during pregnancy on the placental morphology and cytokines.

**Methods:**

Based on Ma’anshan Birth Cohort (MABC) in China, maternal TPOAb levels were retrospectively detected in the first, second and third trimesters. Placental tissues were collected 30 minutes after childbirth, placental morphological indicators were obtained by immediate measurement and formula calculation, and cytokine mRNA expression was detected by real-time quantitative polymerase chain reaction (RT-qPCR) afterward. Generalized linear models and linear mixed models were analyzed for the relationships of maternal TPOAb in the first, second and third trimesters with placental indicators.

**Results:**

Totally 2274 maternal-fetal pairs were included in the analysis of maternal TPOAb levels and placental morphology, and 2122 pairs were included in that of maternal TPOAb levels and placental cytokines. Maternal TPOAb levels in early pregnancy were negatively associated with placental length, thickness, volume, weight and disc eccentricity, while positively correlated with placental IL-6, TNF-α, CRP, CD68, MCP-1, IL-10, HO-1, HIF-1α and GRP78. In mid-pregnancy, maternal TPOAb levels were negatively correlated with placental length, width and area. In late pregnancy, maternal TPOAb levels were negatively correlated with placental length, area, volume and weight. Repeated measures analysis showed that maternal TPOAb positivity tended to increase placental TNF-α, CD68 and MCP-1 while decreasing placental length, width and area than TPOAb negativity. Repeated measures analysis showed that maternal TPOAb levels were positively correlated with placental IL-6, TNF-α, CD68, MCP-1, IL-10, HO-1, HIF-1α and GRP78, while negatively correlated with placental length, area, volume, weight, and disc eccentricity.

**Conclusion:**

There may be trimester-specific associations between maternal TPOAb levels and placental morphology and inflammatory and oxidative stress responses. The effect of maternal TPOAb levels on placental morphology is present throughout pregnancy. Early pregnancy may be the critical period for the association between maternal TPOAb levels and placental inflammatory and oxidative stress responses.

## Introduction

Thyroid peroxidase (TPO) is the primary enzyme involved in producing thyroid hormones (1). Thyroid peroxidase antibody (TPOAb) acts as a competitive inhibitor of the action of TPO and is responsible for thyroid inflammation (2, 3). TPOAb is the most common anti-thyroid autoantibody. TPOAb is a marker of autoimmune thyroid disease (AITD) and would cause thyroid cell damage by activating complement-mediated cytotoxicity and antibody-dependent cell-mediated cytotoxicity (ADCC) (4, 5). TPOAb positivity is frequently present in women of reproductive age. The prevalence of TPOAb positivity observed in pregnant women ranges from 2% to 17%, with a higher prevalence in iodine-deficient populations (6). TPOAb positivity enhances the risk of adverse pregnancy outcomes, including developing thyroid disease during pregnancy, miscarriage, preterm birth, placental abruption, premature rupture of membranes and fetal neurodevelopmental delay (7).

Successful pregnancy maintenance depends on immune homeostasis, immune tolerance and relative cytokines levels (2). Studies have demonstrated that the expression of the inflammatory cytokines tumor necrosis factor-α (TNF-α), interleukin-1β (IL-1β), IL-6, IL-8 and monocyte chemoattractant protein-1 (MCP-1) was elevated in the fetal membranes, cervix, amniotic fluid and placenta (8). TPOAb positivity may lead to failure of immune tolerance at the maternal-fetal interface, severely compromising placental-fetal development (4, 9). Elevated TPOAb levels may modulate immune activity at the cellular level, leading to preterm birth (10). Animal studies indicated extremely preterm birth and fetal intrauterine growth restriction (IUGR) were associated with placental injury and inflammation, as evidenced by the upregulation of inflammatory cytokine and chemokine genes in placenta, including TNF-α, IL-1β, IL-6, MCP-1, macrophage inflammatory peptide-2 (MIP-2) and keratinocyte-derived chemokine (KC) (11). In addition, TPOAb may diffuse through the placental barrier at all stages of pregnancy, increasing immune responses and affecting placental development and pregnancy progression (9, 12). Therefore, it is speculated that maternal TPOAb levels may influence the immune status at the maternal-fetal interface by altering the expression of cytokines in the placenta.

The existence of thyroid autoimmune could indicate a decrease in the ability of the thyroid gland to adapt to the necessary changes related to pregnancy adequately. Hypothyroidism affects fetal-placental development by impairing placental decidualization and vascularization, increasing apoptosis and reducing trophoblast proliferation (13). This may be linked to inflammatory mediators in the placenta. At present, there is only evidence from animal studies suggesting that hypothyroidism affects maternal immune function by interfering with the development of an anti-inflammatory environment, and that maternal hypothyroidism is associated with hypoxia and activation of inflammatory and oxidative stress at the maternal-fetal interface (14, 15). Hypothyroidism reduced the expression of placental interferon-γ (IFN-γ), IL-10 and migration inhibitory factor (MIF) in rats (14), which stimulates the expression of a wide variety of pro-inflammatory cytokines (16). Furthermore, even in the absence of thyroid dysfunction, many studies have linked the presence of TPOAb to adverse maternal-fetal outcomes during pregnancy (9). However, there are no population studies of the association between maternal TPOAb levels and placental cytokines, and it is unclear whether the effect on the placenta is independent or mediated by thyroid hormones.

Placental morphology was significantly associated with placental function, and abnormal placental morphological indicators were related with the increased risk of pregnancy complications (17, 18). In previous studies, limited studies have focused on the effect of maternal TPOAb presence on placental morphology during pregnancy and findings were controversial (19–21). A study from Japan found that placental weight was significantly lower among TPOAb-positive subjects compared with controls mothers (20). However, a cohort study showed that significantly higher placental weights were observed among TPOAb-positive mothers (21). Moreover, evidence from population studies was absent regarding the association of maternal TPOAb levels with placental inflammatory and oxidative stress responses.

In the current study, based on a prospective birth cohort study, we had repeatedly measured maternal TPOAb in the first, second and third trimester of pregnancy. We aimed to examine the relationship of maternal TPOAb levels with placental morphology and inflammatory and oxidative stress responses and to identify the potential critical period.

## Materials and methods

### Participants

This study was based on Ma’anshan Birth Cohort (MABC) in China. Pregnant women who had their first antenatal checkup from May 2013 to September 2014 were invited to join the study. The inclusion criteria were as follows: 1) permanent residents in Ma’anshan City; 2) within 14 weeks of gestation; 3) planning to have pregnancy checkups and delivery at Ma’anshan Maternal and Child Health Center; 4) able to understand and complete the questionnaires (22). Further exclusion criteria were set in the current study as 1) twin pregnancy and adverse pregnancy outcomes (spontaneous abortion, therapeutic abortion, ectopic pregnancy, and stillbirth); 2) having a family history of thyroid diseases and/or those who suffered from thyroid conditions (including hypothyroidism, hyperthyroidism, thyroiditis, thyroid tumor/cancer) before/during pregnancy and received treatment; 3) missing data on maternal TPOAb concentrations; 4) missing data on placental morphology or cytokines.

The current study was a retrospective cohort study from prospectively collected data. Women had been prospectively followed up during pregnancy. After delivery, their children were continuously followed up in the cohort. Maternal thyroid function was determined retrospectively during childhood follow up. Women were not aware of their thyroid function from assays of biological samples collected in this study.

All participants provided written informed consent. The study protocol was approved by the Biomedical Ethics Committee of the Anhui Medical University (No. 20131401).

### Evaluation of maternal thyroid function

Fasting venous blood from women was collected in the first (before 13 weeks), second (14-27 weeks) and third (28 weeks and beyond) trimester of pregnancy, respectively. After centrifugation, the serum was stored at -80°C. During the children’s follow-up period, thyroid-stimulating hormone (TSH), free thyroxine (FT_4_) and TPOAb levels were determined retrospectively by electrochemical immunoassay (Cobas E411 analyzer; Roche Company, Germany). The detection limit of TPOAb was 5.0 IU/mL, and the coefficient of variation between the reagent batches was<10% (22). Women with TPOAb positivity were defined as TPOAb >34.0 IU/mL, otherwise were defined as TPOAb negative controls.

### Measurement of placental indicators

Placenta tissues were collected 30 minutes after delivery, and the placental length, width and thickness were measured immediately. Placental area, volume, weight and disc eccentricity were calculated using the following formulas (23–26).


Placental area = π/4 × placental length × placental width

$$Placental\;volume\;=\;4\pi /3\;\times \;\left(placental\;length\;\times \;placenta\;width\;\times \;placenta\;thickness\right)/2^{3}$$

Placental weight = π/4 × placental length × placental width× placenta thickness

Disc eccentricity = placental length/placental width

Then, the placental lobule without calcification was taken and cut smaller into ≤0.5 cm pieces, and placed in RNAlater solution to allow the solution to thoroughly penetrate the tissue. After removing the supernatant, the tissues were stored at -80°C. Placental cytokines mRNA expression was detected by real-time quantitative polymerase chain reaction (RT-qPCR), including IL-1β, IL-6, TNF-α, IFN-γ, C-reactive protein (CRP), CD68, MCP-1, IL-4, IL-10, heme oxygenase 1 (HO-1), hypoxia-inducible factor 1α (HIF-1α), glucose regulated protein 78 kda (GRP78). The detailed procedures could be found elsewhere in our previous studies (27, 28).

### Covariates

Based on the previous literature and directed acyclic graph (**Supplementary Figure 1**), maternal age, education level, gestational weight gain, monthly income, parity, fetal gender, smoking and drinking during pregnancy were identified as potential confounders. Data on maternal age, education level, monthly income, parity, smoking and drinking during pregnancy were collected questionnaires during recruitment. Information on fetal gender and gestation weight gain was obtained from medical notes.

In addition, TSH and FT_4_ levels, gestational diabetes mellitus (GDM), hypertensive disorder complicating pregnancy (HDCP), maternal infection or inflammation during pregnancy, and gestational age at birth were included in the sensitivity analyses, respectively. Maternal infection or inflammation during pregnancy covered bronchitis, influenza, gastroenteritis, cholecystitis, vaginitis, chorioamnionitis, and pelvic inflammatory disease.

### Statistical analysis

SPSS 26.0 was adopted for statistical analyses and Graphpad Prism 8.0.2 was used for figure drawing. Statistical significance was declared at *P<*0.05.

Compared to the differences between the included and excluded, T-test was used for continuous variables, and chi-square test was performed for categorical variables. Since the distributions of TSH, FT_4_, TPOAb concentrations and placental indicators were right-skewed, they were all ln-transformed. Generalized linear models were analyzed for the relationships of maternal TPOAb exposure in three trimesters with placental indicators, respectively. Linear mixed models, a powerful and robust statistical methods for addressing mixed relationships with the same exposure at different times, were fitted to determine the effect of the repeated measurements of maternal TPOAb on placental indicators.

Five sensitivity analyses were conducted. 1) TPOAb positivity may be related to an increased risk of clinical or subclinical hypothyroidism (29, 30), and maternal hypothyroidism further affects placental growth and development (9, 31). Therefore, thyroid hormone levels may be potential mediators. When analyzing TPOAb in a single trimester, FT_4_ and TSH levels in that trimester and TPOAb levels in the other trimesters were further adjusted. FT_4_ and TSH data during pregnancy were further adjusted when performing repeated measure analysis. 2) Maternal TPOAb may be related to GDM (32). Maternal obesity could influence placental inflammatory status and morphology in human term placenta (33). Maternal GDM was further adjusted. 3) Maternal TPOAb may be related to HDCP (34). HDCP may decrease placental blood flow and oxidative stress (35). Maternal HDCP was further adjusted. 4) Maternal infection or inflammation may cause placental maladjustment (36, 37). Maternal infection or inflammation was further adjusted. 5) The effect of thyroid hormones on placental formation and development varies with gestational age (4). Maternal gestational age was further adjusted.

## Results

### Basic characteristics of participants

**Figure 1:**
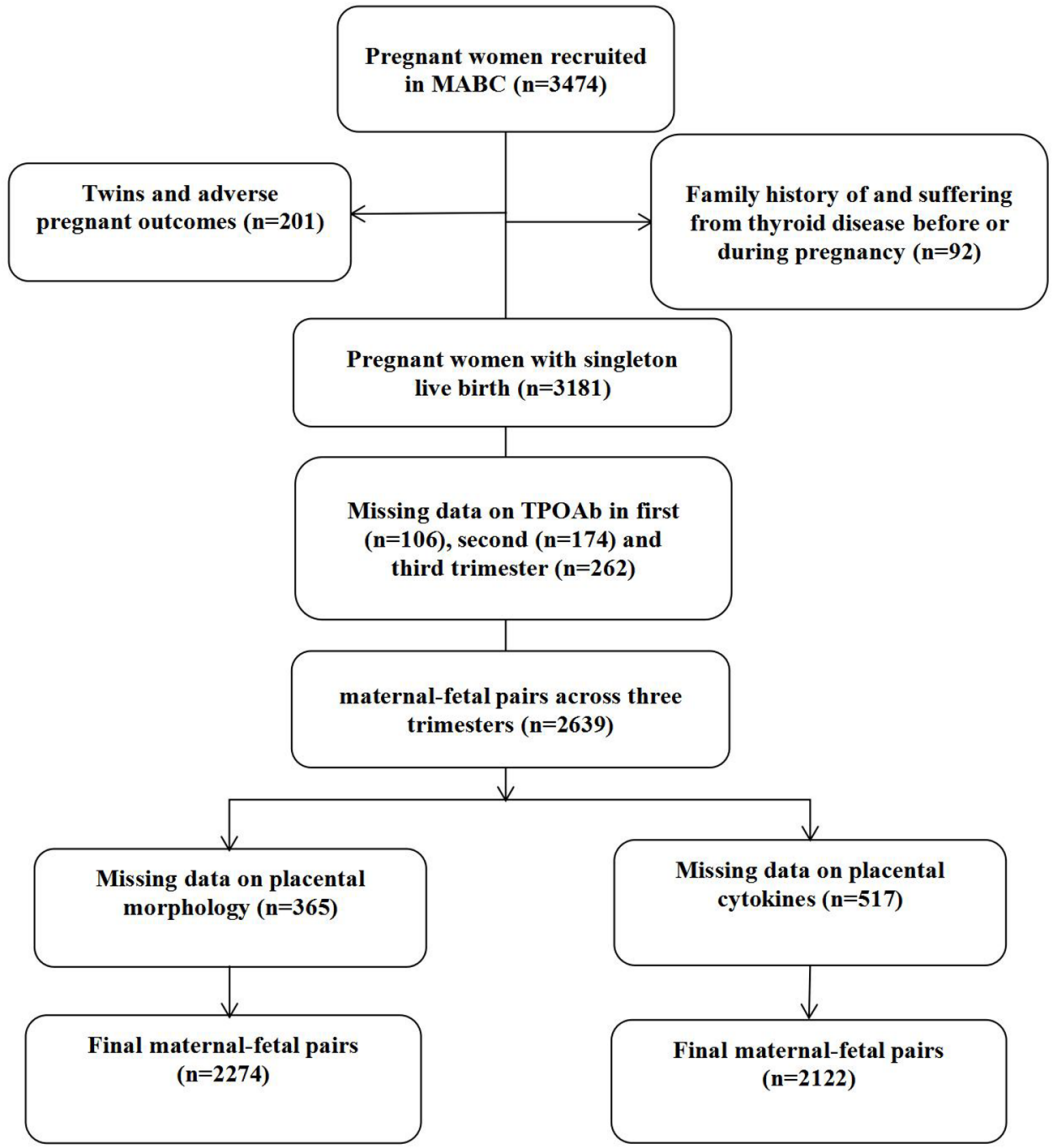
Flowchart of Participants recruitment.

A total of 3474 women were recruited as the initial study population. Totally 2274 maternal-fetal pairs were included in the analysis of maternal TPOAb levels and placental morphology, and 2122 pairs were included in that of maternal TPOAb levels and placental cytokines (**Figure 1**). Baseline characteristics of 2639 included and 835 excluded participants were shown in **Table 1**. Compared with the excluded participants, the included mothers had a higher level of education, a higher gestational age at birth, a lower TSH concentration in the first trimester, a higher percentage of primiparas, a higher prevalence of HDCP and GDM.

**Table 1:** Basic Characteristics of Included and Excluded Participants (n=3474).

Characteristics	Included (n=2639)	Excluded (n=835)	Missing	p-value
Maternal age, year	26.6 ± 3.6	26.9 ± 4.1	0(0)	0.088
Maternal education level,year	13.4 ± 3.1	13.1 ± 3.2	0(0)	0.007
Gestational weight gain, kg	17.9 ± 5.0	17.7 ± 5.3	255(7.3)	0.545
Gestational age at birth, week	39.1 ± 1.2	38.6 ± 1.9	200(5.8)	0.000
Maternal concentrations of TSH during pregnancy, μIU/mL				
In 1st trimester	1.9 ± 2.2	2.4 ± 5.4	143(4.1)	0.047
In 2nd trimester	2.7 ± 1.5	2.8 ± 1.6	346(10.0)	0.298
In 3rd trimester	2.5 ± 1.4	2.5 ± 1.4	520(15.0)	0.652
Maternal concentrations of FT_4_ during pregnancy, pmol/L				
In 1st trimester	17.1 ± 3.3	17.1 ± 5.0	143(4.1)	0.793
In 2nd trimester	12.0 ± 1.7	12.0 ± 1.9	346(10.0)	0.859
In 3rd trimester	13.4 ± 2.4	13.6 ± 2.7	520(15.0)	0.133
Monthly income, yuan			0(0)	0.826
≤2500	703(26.6)	215(25.7)		
2500~4000	1127(42.7)	366(43.8)		
>4000	809(30.7)	254(30.4)		
Parity			0(0)	0.000
Nulliparous	2356(89.3)	707(84.7)		
Multiparous	283(10.7)	128(15.3)		
Maternal smoking			0(0)	0.282
No	2531(95.9)	793(95.0)		
Yes	108(4.1)	42(5.0)		
Maternal drinking			0(0)	0.883
No	2428(92.0)	770(92.2)		
Yes	211(8.0)	65(7.8)		
GDM			153(4.4)	0.000
No	2318(87.8)	561(82.3)		
YES	321(12.2)	121(17.7)		
HDCP			208(6.0)	0.003
No	2489(94.5)	577(91.3)		
YES	145(5.5)	55(8.7)		
Maternal infection or inflammation during pregnancy			201(5.8)	0.628
No	2426(91.9)	579(91.3)		
Yes	213(8.1)	55(8.7)		
Fetal gender			206(5.9)	0.859
Boy	1345(51.0)	325(51.4)		
Girl	1291(49.0)	307(48.6)		

Data are given as Mean ± standard deviations (SD) or n (%). TSH, thyroid stimulating hormone; FT_4_, free thyroxine; GDM, gestational diabetes mellitus; HDCP, hypertensive disorder complicating pregnancy.

### Distribution of maternal TPOAb levels and placental indicators

Maternal TPOAb concentrations during three trimesters, placental morphological indicators and inflammatory and oxidative stress cytokines mRNA expression are presented in **Table 2**. The geometric means of maternal TPOAb concentrations were 19.35, 13.12 and 15.48 IU/mL in the first, second and third trimester of pregnancy, respectively. The positive rates of TPOAb in early, mid, and late pregnancy were 11.6%, 6.7%, and 7.2%, respectively. The overall rate of maternal TPOAb positivity during pregnancy was 12.6%.

**Table 2:** Distribution of maternal TPOAb concentrations, placental morphological indicators, inflammatory and oxidative stress cytokines in the participants.

	GM	P25	P50	P75
TPOAb concentration (IU/mL, n=2639)				
First trimester	19.35	12.97	19.29	24.66
Second trimester	13.12	9.00	12.42	17.01
Third trimester	15.48	11.19	14.78	19.63
Placental size (n=2274)				
Placental length (cm)	18.87	17.60	18.50	20.00
Placental width (cm)	16.52	15.50	16.50	17.80
Placental thickness (cm)	2.31	2.00	2.30	2.60
Placental area (cm^2^)	244.72	219.80	240.20	275.54
Placental volume (cm^3^)	376.21	315.62	377.26	456.44
Placental weight (g)	564.32	473.43	565.89	684.65
Disc eccentricity	1.14	1.06	1.11	1.19
Placental cytokines mRNA expression (n=2122)				
IL-1β	2.46	0.93	2.45	6.74
IL-6	2.22	0.98	2.20	5.67
TNF-α	4.26	1.56	4.52	12.70
IFN-γ	3.16	0.95	3.43	11.08
CRP	3.62	0.83	4.09	20.27
CD68	6.92	1.57	7.92	30.56
MCP-1	1.68	0.70	1.78	4.25
IL-4	2.43	0.93	2.18	6.61
IL-10	2.84	1.00	2.55	8.27
HO-1	2.75	1.16	2.82	6.54
HIF-1α	1.69	0.69	1.65	4.31
GRP78	3.57	0.90	3.80	18.07

GM, geometric mean.

### Association between maternal TPOAb and placental morphology

In early pregnancy, TPOAb levels were negatively correlated with placental length (β -0.006, 95%CI -0.011 to -0.001), thickness (β -0.010, 95%CI -0.020 to -0.0004), volume (β -0.016, 95%CI -0.029 to -0.002), weight (β -0.016, 95%CI -0.029 to -0.002) and disc eccentricity (β -0.007, 95%CI -0.012 to -0.002). In the second trimester, TPOAb levels were negatively correlated with placental length (β -0.007, 95%CI -0.013 to -0.001), width (β -0.007, 95%CI -0.014 to -0.00001) and area (β -0.014, 95%CI -0.025 to -0.002). In late pregnancy, TPOAb levels were negatively correlated with placental length (β -0.011, 95%CI -0.018 to -0.003), area (β -0.017, 95%CI -0.030 to -0.005), volume (β -0.019, 95%CI -0.038 to -0.0002) and weight (β -0.019, 95%CI -0.038 to -0.0002) (**Table 3**).

**Table 3:** Association (β and 95% confidence intervals) of maternal TPOAb exposure (IU/mL) and placental morphological indicators (n=2274).

	Placental Length	Placental Width	Placental Thickness	Placental Area	Placental Volume	Placental Weight	Disc Eccentricity
TPOAb_a	-0.006 (-0.011,-0.001)*	0.001 (-0.005,0.006)	-0.010 (-0.020,-0.0004)*	-0.005 (-0.015,0.004)	-0.016 (-0.029,-0.002)*	-0.016 (-0.029,-0.002)*	-0.007 (-0.012,-0.002)*
TPOAb_b	-0.007 (-0.013,-0.001)*	-0.007 (-0.014,-0.00001)*	0.010 (-0.002,0.022)	-0.014 (-0.025,-0.002)*	-0.004 (-0.021,0.013)	-0.004 (-0.021,0.013)	-0.00007 (-0.007,0.007)
TPOAb_c	-0.011 (-0.018,-0.003)**	-0.007 (-0.015,0.001)	-0.002 (-0.015,0.012)	-0.017 (-0.030,-0.005)**	-0.019 (-0.038,-0.0002)*	-0.019 (-0.038, -0.0002)*	-0.004 (-0.011,0.004)

Adjusted for maternal age, maternal education level, gestational weight gain, monthly income, parity, smoking, drinking and fetal gender.

a: First Trimester; b: Second Trimester; c: Third Trimester.

*: P< 0.05. **: P< 0.01.

**Figure 2:**
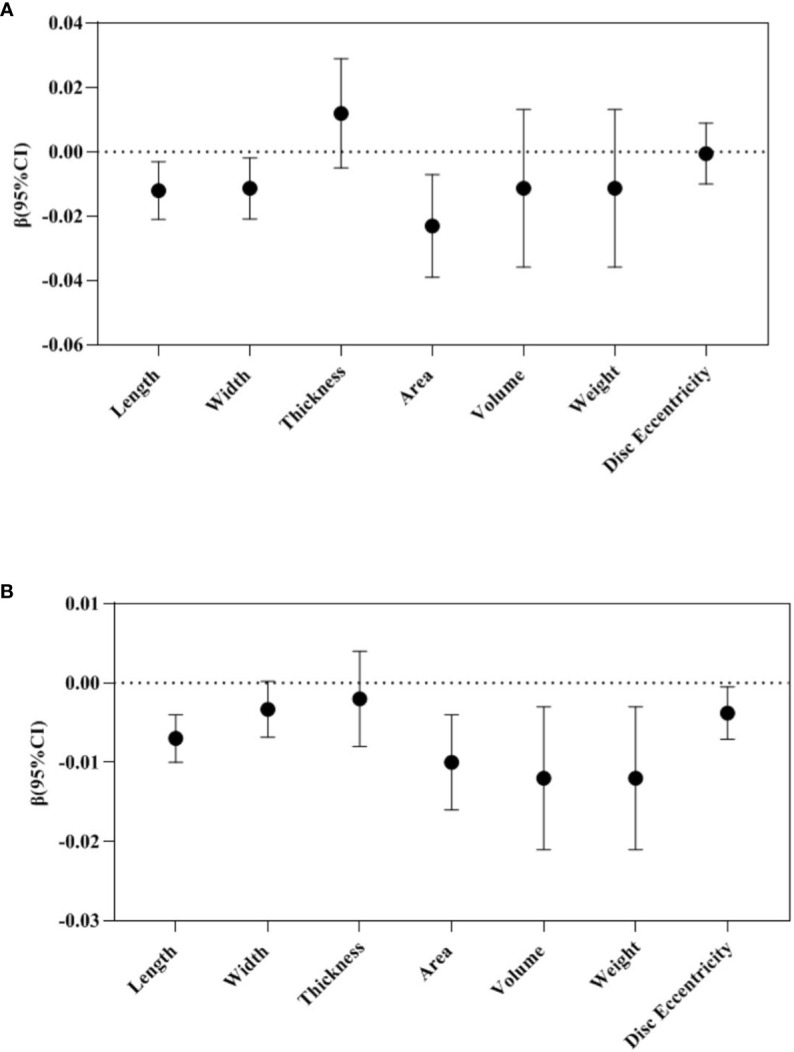
Repeated measure analysis: Association between maternal TPOAb exposure and placental morphology (n=2274). **(A)** Maternal TPOAb positive (vs TPOAb negative) increased placental length, width and area. **(B)** Maternal TPOAb levels throughout pregnancy were negatively associated with placental length, area, volume, weight and disc eccentricity. Adjusted for maternal age, maternal education years, gestational weight gain, monthly income, parity, smoking, drinking and fetal gender.

In the repeated measures analysis, maternal TPOAb positivity during pregnancy tended to reduce placental length (β -0.012, 95%CI -0.021 to -0.003), width (β -0.011, 95%CI -0.021 to -0.002), and area (β -0.023, 95%CI -0.039 to -0.007) (**Figure 2**).

In the repeated measures analysis, maternal TPOAb levels during pregnancy were negatively correlated with placental length (β -0.007, 95%CI -0.010 to -0.004), area (β -0.010, 95%CI -0.016 to -0.004), volume (β -0.012, 95%CI -0.021 to -0.003), weight (β -0.012, 95%CI -0.021 to -0.003) and disc eccentricity (β -0.004, 95%CI -0.007 to -0.0004) (**Figure 2**).

### Association between maternal TPOAb and placental cytokines

In early pregnancy, TPOAb levels were positively correlated with placental IL-6 (β 0.193, 95%CI 0.127-0.259), TNF-α (β 0.161, 95%CI 0.086-0.236), CRP (β 0.167, 95%CI 0.055-0.279), CD68 (β 0.370, 95%CI 0.276-0.464), MCP-1 (β 0.198, 95%CI 0.131-0.265), IL-10 (β 0.143, 95%CI 0.065-0.221), HO-1 (β 0.169, 95%CI 0.098-0.240), HIF-1α (β 0.176, 95%CI 0.105-0.246), GRP78 (β 0.286, 95%CI 0.189-0.383) mRNA expression (**Table 4**).

**Table 4:** Association (β and 95% confidence intervals) of maternal TPOAb exposure (IU/mL) and placental inflammatory and oxidative stress cytokines (n=2122).

	IL-1β	IL-6	TNF-α	IFN-γ	CRP	CD68	MCP-1	IL-4	IL-10	HO-1	HIF-1α	GRP78
TPOAb_a	0.066 (-0.005, 0.138)	0.193 (0.127, 0.259)**	0.161 (0.086, 0.236)**	0.034 (-0.053, 0.121)	0.167 (0.055, 0.279)**	0.370 (0.276, 0.464)**	0.198 (0.131, 0.265)**	0.002 (-0.074, 0.077)	0.143 (0.065, 0.221)**	0.169 (0.098, 0.240)**	0.176 (0.105, 0.246)**	0.286 (0.189, 0.383)**
TPOAb_b	-0.051 (-0.139, 0.036)	0.049 (-0.033, 0.131)	0.058 (-0.034, 0.150)	0.080 (-0.027, 0.187)	-0.033 (-0.170, 0.105)	0.106 (-0.011, 0.223)	-0.002 (-0.085, 0.081)	0.047 (-0.045, 0.139)	-0.009 (-0.105, 0.087)	-0.032 (-0.119, 0.055)	-0.074 (-0.161, 0.013)	-0.078 (-0.198, 0.043)
TPOAb_c	-0.050 (-0.149, 0.048)	0.020 (-0.072, 0.112)	0.049 (-0.054, 0.153)	0.043 (-0.077, 0.163)	-0.004 (-0.159, 0.151)	0.096 (-0.035, 0.228)	0.030 (-0.063, 0.123)	0.052 (-0.051, 0.156)	0.062 (-0.047, 0.170)	0.011 (-0.087, 0.109)	-0.007 (-0.105, 0.090)	-0.007 (-0.142, 0.129)

Adjusted for maternal age, maternal education level, gestational weight gain, monthly income, parity, smoking, drinking and fetal gender.

a: First Trimester; b: Second Trimester; c: Third Trimester.

**: P< 0.01.

**Figure 3:**
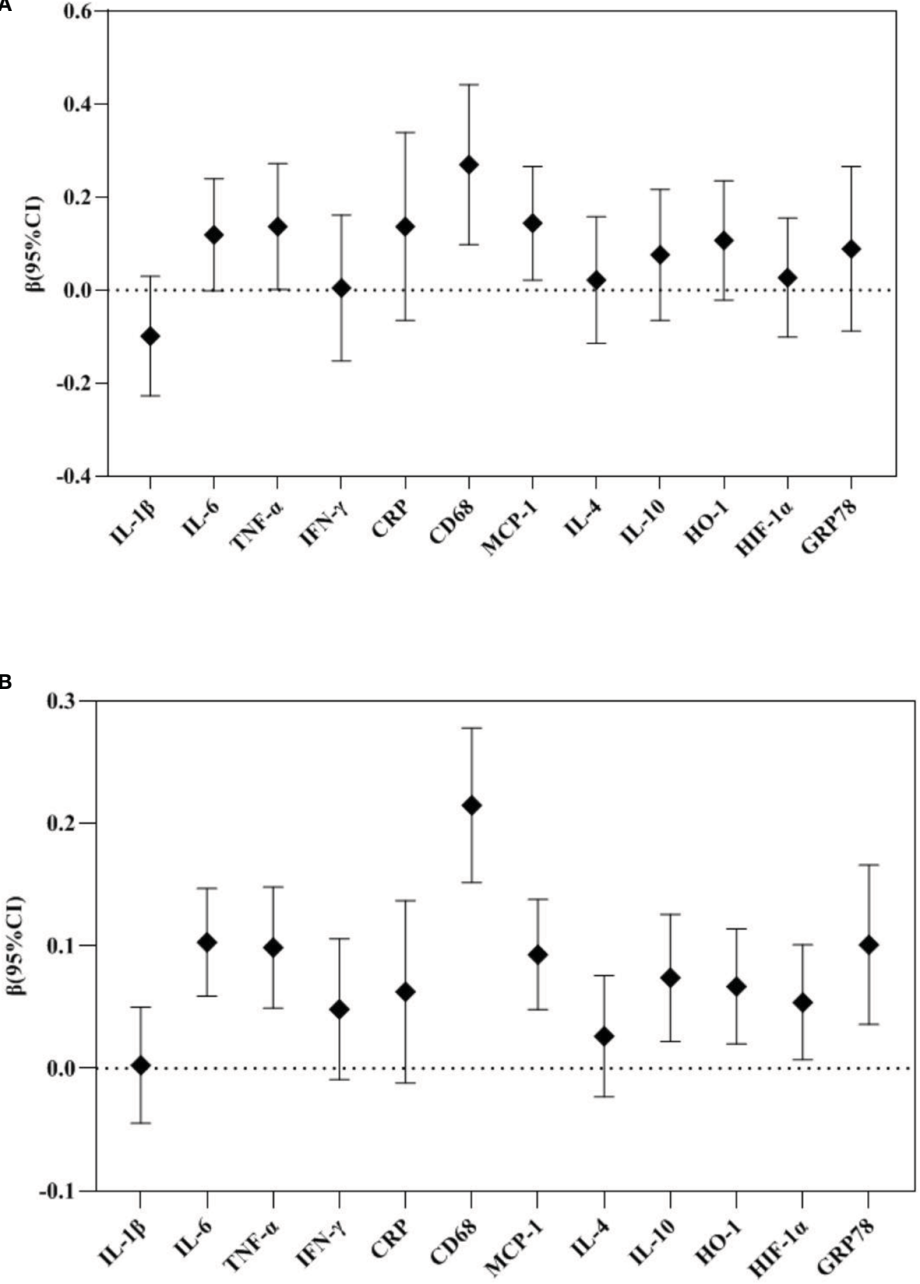
Repeated measure analysis: Association between maternal TPOAb exposure and placental cytokines (n=2122). **(A)** Maternal TPOAb positive (vs TPOAb negative) increased placental TNF-α, CD68 and MCP-1. **(B)** Maternal TPOAb levels throughout pregnancy were negatively associated with placental IL-6, TNF-α, CD68, MCP-1, IL-10, HO-1, HIF-1α and GRP78. Adjusted for maternal age, maternal education years, gestational weight gain, monthly income, parity, smoking, drinking and fetal gender.

In the repeated measures analysis, maternal TPOAb positivity tended to increase placental TNF-α (β 0.137, 95%CI 0.002-0.272), CD68 (β 0.270, 95%CI 0.098-0.442) and MCP-1 (β 0.144, 95%CI 0.022-0.266) mRNA expression (**Figure 3**).

In the repeated measures analysis, maternal TPOAb levels during pregnancy were positively correlated with placental IL-6 (β 0.103, 95%CI 0.059-0.147), TNF-α (β 0.099, 95%CI 0.049-0.148), CD68 (β 0.215, 95%CI 0.152-0.278), MCP-1 (β 0.093, 95%CI 0.048-0.138), IL-10 (β 0.074, 95%CI 0.022-0.126), HO-1 (β 0.067, 95%CI 0.020-0.114), HIF-1α (β 0.054, 95%CI 0.007-0.101), GRP78 (β 0.101, 95%CI 0.036-0.166) mRNA expression (**Figure 3**).

Sensitivity analyses did not fundamentally change the results of the main analyses, and details of the repeated measures and sensitivity analyses are provided in the **Supplementary Tables 1**–**6**.

## Discussion

In this prospective cohort study, maternal TPOAb levels were found to be negatively related to placental morphology in the first, second and third trimester of pregnancy. Maternal TPOAb positivity tended to decrease placental length, width and area. The current study also provides longitudinal evidence that maternal exposure to TPOAb during pregnancy may be an important risk factor for maternal-placental immune activation. Significant positive associations between maternal TPOAb levels and placental mRNA expression of IL-6, TNF-α, CRP, CD68, MCP-1, IL-10, HO-1, HIF-1α and GRP78 in early pregnancy were observed. Maternal TPOAb positivity tended to increase TNF-α, CD68 and MCP-1 mRNA expression.

Altered placental morphology may reflect its responses to intrauterine stress during early placental development (38). Our results are consistent with previous studies showing lower placental morphological indicators in TPOAb-positive mothers than in control women. Spinillo et al. found that TPOAb-positive women with TSH ≥2.5 mU/L had lower placental volume and area than euthyroid TPOAb-negative women (19). Tissues growing along the length and width of the placenta have different functions and are influenced by different factors. Tissues growing across the width could be involved in maternal-fetal nutrient transport. However, the tissues along the length have different functions but remain to be studied (39, 40). Placental thickness is the main dimension of placental growth in late pregnancy and may reflect the vascularization of the chorionic villi. Placental surface area is mainly established before late pregnancy and may indicate the number of spiral arteries supplying the placenta (38, 41).

Placental weight can be used as a proxy for fetal metabolic rate. In our study, maternal TPOAb levels were negatively correlated with placental weight. Recently, there have been conflicting studies on the association between TPOAb levels and placental weight. A small retrospective study from Japan showed that placental weight was lower in TPOAb-positive women (20). Männistö et al. found that significantly higher placental weights were observed in TPOAb-positive mothers as well as women with high TSH and low FT_4_ levels in early pregnancy based on the Northern Finland Birth Cohort (21). An animal study showed that hypothyroidism reduced placental weight in rats (42). Placental weight and disc eccentricity were reported to be directly related to placental stress (43). This suggests maternal TPOAb levels may directly affect placentation and impair placental growth and development.

The negative correlation between maternal TPOAb levels and placental morphology in our study was present in all three trimesters, suggesting that maternal TPOAb levels during pregnancy may be an essential risk factor for placental development. Variation in placental morphology is common, but this variation must be limited to a specific range to ensure adequate placental function. The greater the degree of variability in placental morphology, the more severe the decline in function.

Abnormal expression of placental inflammatory and oxidative stress cytokines may respond to high levels of maternal TPOAb. In this study, rising maternal TPOAb levels correlated with an upward trend in inflammatory cytokines. Early pregnancy is the critical period for this association. Increased numbers of Th1 and decreased frequencies of Th2 in the endometrial leukocyte of women with autoimmune thyroid disease would lead to hypersecretion of IFN-γ and reduced production of IL-4 and IL-10 (44). Related studies have also shown that the ratio of TNF-α/IL-10 produced by CD3+/CD4+ cells is significantly higher in women with AITD compared to normal controls (45). This suggests that the maternal TPOAb levels moderately aggravate placental inflammatory status and interfere with placental immune tolerance.

There is a unique immunologic state during successful pregnancy, the balance between the anti-inflammatory and pro-inflammatory environments changes to facilitate the establishment and maintenance of immune tolerance and promote placental development, but excessive inflammation can affect placental function (2, 46). Abnormal expression of placental cytokines may lead to insufficiency and increase the risk of adverse pregnancy outcomes, such as pre-eclampsia (PE) and preterm birth (47, 48). Ma et al. found higher expression of placental pro-inflammatory cytokines IL-1β, IL-6 and MCP-1 in pregnant women with PE (49). Animal studies have shown that exacerbated levels of IL-6 and TNF-α contribute to placental dysfunction (50). Excessive TNF-α in the placenta may impair trophoblast fusion and hormone production and promote apoptosis (51, 52). CD68, primarily localized to lysosomes and endosomes, could be significantly upregulated in macrophages in response to inflammatory stimuli (53). Kim et al. found that maternal CRP was deposited in the human placenta, and its elevated level was related to chorioamnionitis, PE and preterm birth (54).

The maintenance of immune tolerance during pregnancy depends on the expression of anti-inflammatory cytokines, which is critical to prevent fetal immune rejection (55). In our study, TPOAb levels were found to be positively correlated with placental IL-10 mRNA expression in early pregnancy. IL-10 suppresses the production of many pro-inflammatory cytokines, such as IL-6 and TNF-α. IL-10 promotes trophoblast differentiation, inhibits trophoblast invasion and indirectly stimulates angiogenesis (46). It is suggested that increased IL-10 expression may be involved in regulating the adverse effects of inflammation and oxidative stress, which may be a protective mechanism of the placenta against high TPOAb levels.

This study firstly reported that high levels of TPOAb may be associated with elevated placental oxidative stress cytokines. An animal study had shown that hypothyroidism decreased the expression of HO-1, GRP78 and C/EBP homologous protein (CHOP) genes/proteins in the mid-gestation while increased the expression of HIF-1α, CHOP and nuclear factor erythroid 2-related factor 2 (NRF2) genes/proteins (15). HO-1 is a crucial cytokine for immune tolerance and promotes the establishment of an anti-inflammatory environment (56). HO-1 expression is induced under hypoxic conditions and modulates IL-10 signaling (57). HIF-1α is a key regulator of the cellular response to the hypoxic environment and plays a critical role in regulating trophoblast differentiation and invasion and in spiral artery remodeling (58). CHOP and GRP78 are known endoplasmic reticulum stress markers, and their elevation promotes trophoblast fusion, syncytialization and invasion (59–61). Spinillo et al. found that in women with positive TPOAb in early pregnancy, the presence of multiple placental pathological features suggested placental hypoxic/ischemic injury (38, 62). This suggests that high levels of TPOAb may activate oxidative stress at the maternal-fetal interface.

This study has several strengths. To the best of our knowledge, this is the first study to systematically investigate the potential effect of maternal TPOAb levels in the first, second and third trimester of pregnancy on placental morphology and inflammatory and oxidative stress responses based on a prospective birth cohort study. All information about exposures, outcomes and potential confounding factors was collected prospectively, effectively avoiding recall and confounding bias. Second, thyroid indicators, including TPOAb levels, were measured retrospectively during childhood follow-up period. Women did not know their thyroid function from our biological samples. Except for those who might have had interventions during routine antenatal checkups (and those women were actually excluded from the current study), women did not have any interventions or medications relevant to thyroid function. This would provide natural and real data on the thyroid function of the participants during pregnancy. Actually, studies had revealed that levothyroxine supplementation in TPO euthyroid women was not associated with adverse perinatal outcomes in TPO-positive women with normal thyroid function (63). Third, multidimensional placental indicators were used to provide a comprehensive measurement of placental morphology and inflammatory and oxidative stress responses. This allowed a wide understanding on the effect of maternal TPOAb levels on placental morphology and function. Furthermore, multiple sensitivity analyses were performed, fully considering maternal conditions, including GDM, HDCP and systemic inflammation. After further adjusting for these variables, the main findings remained unchanged. It highly increases the robustness and precision of the findings, and indicates that the potential effect of maternal TPOAb exposure on placental morphology and inflammation and oxidative stress may be independent of maternal systemic endocrinal or inflammatory conditions.

Some limitations must be recognized. Firstly, only data on the expression of placental transcriptional biomarkers are available in this study, which only indirectly reflects protein translation. But mRNA assay is more feasible than protein levels in a large sample studies measuring multiple cytokines. Secondly, human placenta samples can usually be collected after childbirth. Dynamic placental monitoring can better reflect placental temporal and spatial characteristics, which could be observed by antenatal ultrasound scan. However, in the current study, we did not have data on maternal ultrasound scan. Thirdly, information on local basic iodine levels needed to be in this study, as it would be an important factor related to individual thyroid function. However, Ma’anshan City has yet to be reported as an iodine area. Finally, although many potential confounders were considered in the current study, residual confounding factors could not be ruled out, such as maternal exposure to selenium (64), heavy metals (11, 65), and environmental endocrine disruptors (66).

## Conclusions

In conclusion, there may be trimester-specific associations between maternal TPOAb levels and placental morphology and inflammatory and oxidative stress responses. The effect of maternal TPOAb levels on placental morphology is present throughout pregnancy. Early pregnancy may be the critical period for the association between maternal TPOAb levels and placental inflammatory and oxidative stress responses. This study provides evidence for the potentially independent effect of maternal TPOAb on placental morphology and function, which act as the pathway to pregnancy outcomes. Close monitoring of women’s TPOAb levels during pregnancy is believable to be important for placental development and subsequent fetal health.

## Data availability statement

The original contributions presented in the study are included in the article/**Supplementary Material**, further inquiries can be directed to the corresponding author.

## Ethics statement

The studies involving humans were approved by Biomedical Ethics Committee of the Anhui Medical University (No. 20131401). The studies were conducted in accordance with the local legislation and institutional requirements. The participants provided their written informed consent to participate in this study

## Author contributions

XR and KH performed data analysis and wrote the paper. MY and YT conceived study design and performed data analysis. YH, YBH and JW conducted data acquisition and interpretation and laboratory testing. FT provided project administration and resource support. KH obtained funding for this research. All authors contributed to the article and approved the submitted version.

## References

[1] Trbojević B Djurica S. Srp arh celok lek. Srpski arhiv za celokupno lekarstvo (2005) 133(Suppl 1):25–33. doi: 10.2298/sarh05s1025t 16405253

[2] Löb S Amann N Kuhn C Schmoeckel E Wöckel A Zati Zehni A. Interleukin-1 beta is significantly upregulated in the decidua of spontaneous and recurrent miscarriage placentas. J Reprod Immunol (2021) 144:103283. doi: 10.1016/j.jri.2021.103283 33545613

[3] Ferrari SM Paparo SR Ragusa F Elia G Mazzi V Patrizio A. Chemokines in thyroid autoimmunity. Best Pract Res Clin Endocrinol Metab (2023) 37(2):101773. doi: 10.1016/j.beem.2023.101773 36907786

[4] Springer D Jiskra J Limanova Z Zima T Potlukova E. Thyroid in pregnancy: From physiology to screening. Crit Rev Clin Lab Sci (2017) 54(2):102–16. doi: 10.1080/10408363.2016.1269309 28102101

[5] Chardès T Chapal N Bresson D Bès C Giudicelli V Lefranc MP. The human anti-thyroid peroxidase autoantibody repertoire in Graves' and Hashimoto's autoimmune thyroid diseases. Immunogenetics (2002) 54(3):141–57. doi: 10.1007/s00251-002-0453-9 12073143

[6] Moreno-Reyes R Glinoer D Van Oyen H Vandevijvere S. High prevalence of thyroid disorders in pregnant women in a mildly iodine-deficient country: a population-based study. J Clin Endocrinol Metab (2013) 98(9):3694–701. doi: 10.1210/jc.2013-2149 23846819

[7] Dhillon-Smith RK Coomarasamy A. TPO antibody positivity and adverse pregnancy outcomes. Best Pract Res Clin Endocrinol Metab (2020) 34(4):101433. doi: 10.1016/j.beem.2020.101433 32883611

[8] Chen CY Chen CP Lin KH. Biological functions of thyroid hormone in placenta. Int J Mol Sci (2015) 16(2):4161–79. doi: 10.3390/ijms16024161 PMC4346950 25690032

[9] Vissenberg R Manders VD Mastenbroek S Fliers E Afink GB Ris-Stalpers C. Pathophysiological aspects of thyroid hormone disorders/thyroid peroxidase autoantibodies and reproduction. Hum Reprod Update (2015) 21(3):378–87. doi: 10.1093/humupd/dmv004 25634660

[10] Kyrilli A Unuane D Poppe KG. Thyroid autoimmunity and pregnancy in euthyroid women. Best Pract Res Clin Endocrinol Metab (2023) 37(2):101632. doi: 10.1016/j.beem.2022.101632 35256265

[11] Zhu YD Liang CM Hu YB Li ZJ Wang SF Xiang HY. Repeated measures of prenatal thallium exposure and placental inflammatory cytokine mRNA expression: The Ma'anshan birth cohort (MABC) study. Chemosphere (2020) 246:125721. doi: 10.1016/j.chemosphere.2019.125721 31911326

[12] Abdolmohammadi-Vahid S Samaie V Hashemi H Mehdizadeh A Dolati S Ghodrati-Khakestar F. Anti-thyroid antibodies and underlying generalized immunologic aberrations in patients with reproductive failures. J Reprod Immunol (2022) 154:103759. doi: 10.1016/j.jri.2022.103759 36332368

[13] Silva JF Ocarino NM Serakides R. Thyroid hormones and female reproduction. Biol Reprod (2018) 99(5):907–21. doi: 10.1093/biolre/ioy115 29767691

[14] Silva JF Ocarino NM Serakides R. Maternal thyroid dysfunction affects placental profile of inflammatory mediators and the intrauterine trophoblast migration kinetics. Reproduction (2014) 147(6):803–16. doi: 10.1530/REP-13-0374 24534949

[15] Dos Anjos Cordeiro JM Santos LC de Oliveira LS Santos BR Santos EO Barbosa EM. Maternal hypothyroidism causes oxidative stress and endoplasmic reticulum stress in the maternal-fetal interface of rats. Free Radic Biol Med (2022) 191:24–39. doi: 10.1016/j.freeradbiomed.2022.08.033 36038036

[16] Cardaropoli S Paulesu L Romagnoli R Ietta F Marzioni D Castellucci M. Macrophage migration inhibitory factor in fetoplacental tissues from preeclamptic pregnancies with or without fetal growth restriction. Clin Dev Immunol (2012) 2012:639342. doi: 10.1155/2012/639342 22007254 PMC3189467

[17] Takemoto R Anami A Koga H. Relationship between birth weight to placental weight ratio and major congenital anomalies in Japan. PloS One (2018) 13(10):e0206002. doi: 10.1371/journal.pone.0206002 30346975 PMC6197685

[18] Frayne J Nguyen T Hauck Y Liira H Keelan JA. The relationship between pregnancy exposure to antidepressant and atypical antipsychotic medications and placental weight and birth weight ratio: A retrospective cohort study. J Clin Psychopharmacol (2018) 38(6):563–9. doi: 10.1097/JCP.0000000000000964 30346334

[19] Spinillo A De Maggio I Ruspini B Bellingeri C Cavagnoli C Giannico S. Placental pathologic features in thyroid autoimmunity. Placenta (2021) 112:66–72. doi: 10.1016/j.placenta.2021.07.287 34304015

[20] Ushijima J Furukawa S Sameshima H. The presence of thyroid peroxidase antibody is associated with lower placental weight in maternal thyroid dysfunction. Tohoku J Exp Med (2019) 249(3):231–6. doi: 10.1620/tjem.249.231 31776300

[21] Männistö T Vääräsmäki M Pouta A Hartikainen AL Ruokonen A Surcel HM. Perinatal outcome of children born to mothers with thyroid dysfunction or antibodies: a prospective population-based cohort study. J Clin Endocrinol Metab (2009) 94(3):772–9. doi: 10.1210/jc.2008-1520 19106271

[22] Li P Teng Y Ru X Liu Z Han Y Tao F. Sex-specific effect of maternal thyroid hormone trajectories on preschoolers' Behavioral development: A birth cohort study. J Clin Endocrinol Metab (2022) 107(5):e2037–46. doi: 10.1210/clinem/dgab887 34999790

[23] Burton GJ Fowden AL Thornburg KL. Placental origins of chronic disease. Physiol Rev (2016) 96(4):1509–65. doi: 10.1152/physrev.00029.2015 PMC5504455 27604528

[24] Barker DJP Eriksson JG Kajantie E Alwasel SH Fall CHD Roseboom TJ. The maternal and placental origins of chronic disease. In: Burton GJ Barker DJP Moffett A Thornburg K, editors. The Placenta and Human Developmental Programming. Cambridge: Cambridge University Press (2010). p. 5–16.

[25] Barker DJ Bull AR Osmond C Simmonds SJ. Fetal and placental size and risk of hypertension in adult life. BMJ (1990) 301(6746):259–62. doi: 10.1136/bmj.301.6746.259 PMC1663477 2390618

[26] Punshon T Li Z Jackson BP Parks WT Romano M Conway D. Placental metal concentrations in relation to placental growth, efficiency and birth weight. Environ Int (2019) 126:533–42. doi: 10.1016/j.envint.2019.01.063 PMC6475117 30851484

[27] Hu Y Huang K Sun Y Wang J Xu Y Yan S. Placenta response of inflammation and oxidative stress in low-risk term childbirth: the implication of delivery mode. BMC Pregnancy Childbirth (2017) 17(1):407. doi: 10.1186/s12884-017-1589-9 29207957 PMC5718001

[28] Zhou J Teng Y Zhang F Ru X Li P Wang J. Sex-specific association between placental inflammatory cytokine mRNA expression and preschoolers' behavioral development: The Ma'anshan birth cohort study. Brain Behav Immun (2022) 104:110–21. doi: 10.1016/j.bbi.2022.05.017 35661681

[29] Sieiro Netto L Medina Coeli C Micmacher E Mamede Da Costa S Nazar L Galvão D. Influence of thyroid autoimmunity and maternal age on the risk of miscarriage. Am J Reprod Immunol (2004) 52(5):312–6. doi: 10.1111/j.1600-0897.2004.00227.x 15550067

[30] Sun J Teng D Li C Peng S Mao J Wang W. Association between iodine intake and thyroid autoantibodies: a cross-sectional study of 7073 early pregnant women in an iodine-adequate region. J Endocrinol Invest (2020) 43(1):43–51. doi: 10.1007/s40618-019-01070-1 31264141

[31] Adu-Gyamfi EA Wang YX Ding YB. The interplay between thyroid hormones and the placenta: a comprehensive review†. Biol Reprod (2020) 102(1):8–17. doi: 10.1093/biolre/ioz182 31494673

[32] Karakosta P Alegakis D Georgiou V Roumeliotaki T Fthenou E Vassilaki M. Thyroid dysfunction and autoantibodies in early pregnancy are associated with increased risk of gestational diabetes and adverse birth outcomes. J Clin Endocrinol Metab (2012) 97(12):4464–72. doi: 10.1210/jc.2012-2540 23015651

[33] Nogues P Dos Santos E Couturier-Tarrade A Berveiller P Arnould L Lamy E. Maternal obesity influences placental nutrient transport, inflammatory status, and morphology in human term placenta. J Clin Endocrinol Metab (2021) 106(4):e1880–96. doi: 10.1210/clinem/dgaa660 32936881

[34] Alavi A Adabi K Nekuie S Jahromi EK Solati M Sobhani A. Thyroid dysfunction and autoantibodies association with hypertensive disorders during pregnancy. J Pregnancy (2012) 2012:742695. doi: 10.1155/2012/742695 22848832 PMC3405662

[35] Naruse K Tsunemi T Kawahara N Kobayashi H. Preliminary evidence of a paternal-maternal genetic conflict on the placenta: Link between imprinting disorder and multi-generational hypertensive disorders. Placenta (2019) 84:69–73. doi: 10.1016/j.placenta.2019.02.009 30846225

[36] Goldstein JA Gallagher K Beck C Kumar R Gernand AD. Maternal-fetal inflammation in the placenta and the developmental origins of health and disease. Front Immunol (2020) 11:531543. doi: 10.3389/fimmu.2020.531543 33281808 PMC7691234

[37] Harding AT Goff MA Froggatt HM Lim JK Heaton NS. GPER1 is required to protect fetal health from maternal inflammation. Science (2021) 371(6526):271–6. doi: 10.1126/science.aba9001 PMC8060949 33446553

[38] Freedman AA Hogue CJ Marsit CJ Rajakumar A Smith AK Goldenberg RL. Associations between the features of gross placental morphology and birthweight. Pediatr Dev Pathol (2019) 22(3):194–204. doi: 10.1177/1093526618789310 30012074 PMC6335186

[39] Eriksson JG Kajantie E Thornburg KL Osmond C Barker DJ. Mother's body size and placental size predict coronary heart disease in men. Eur Heart J (2011) 32(18):2297–303. doi: 10.1093/eurheartj/ehr147 PMC3697804 21632601

[40] Kajantie E Thornburg KL Eriksson JG Osmond C Barker DJ. In preeclampsia, the placenta grows slowly along its minor axis. Int J Dev Biol (2010) 54(2-3):469–73. doi: 10.1387/ijdb.082833ek 19876819

[41] Salafia CM Maas E Thorp JM Eucker B Pezzullo JC Savitz DA. Measures of placental growth in relation to birth weight and gestational age. Am J Epidemiol (2005) 162(10):991–8. doi: 10.1093/aje/kwi305 16192346

[42] Silva JF Vidigal PN Galvão DD Boeloni JN Nunes PP Ocarino NM. Fetal growth restriction in hypothyroidism is associated with changes in proliferative activity, apoptosis and vascularisation of the placenta. Reprod Fertil Dev (2012) 24(7):923–31. doi: 10.1071/RD11219 22935153

[43] Yampolsky M Salafia CM Shlakhter O Haas D Eucker B Thorp J. Modeling the variability of shapes of a human placenta. Placenta (2008) 29(9):790–7. doi: 10.1016/j.placenta.2008.06.005 PMC2570048 18674815

[44] Stewart-Akers AM Krasnow JS Brekosky J Deloia JA. Endometrial leukocytes are altered numerically and functionally in women with implantation defects. Am J Reprod Immunol (1998) 39(1):1–11. doi: 10.1111/j.1600-0897.1998.tb00326.x 9458927

[45] Kim NY Cho HJ Kim HY Yang KM Ahn HK Thornton S. Thyroid autoimmunity and its association with cellular and humoral immunity in women with reproductive failures. Am J Reprod Immunol (2011) 65(1):78–87. doi: 10.1111/j.1600-0897.2010.00911.x 20712806

[46] Vasilopoulou E Loubière LS Lash GE Ohizua O McCabe CJ Franklyn JA. Triiodothyronine regulates angiogenic growth factor and cytokine secretion by isolated human decidual cells in a cell-type specific and gestational age-dependent manner. Hum Reprod (2014) 29(6):1161–72. doi: 10.1093/humrep/deu046 PMC4017942 24626803

[47] Wen Y Cheng M Qin L Xu W. TNFα-induced abnormal activation of TNFR/NF-κB/FTH1 in endometrium is involved in the pathogenesis of early spontaneous abortion. J Cell Mol Med (2022) 26(10):2947–58. doi: 10.1111/jcmm.17308 PMC9097845 35441429

[48] Fakhr Y Koshti S Habibyan YB Webster K Hemmings DG. Tumor necrosis factor-α Induces a preeclamptic-like phenotype in placental villi via sphingosine kinase 1 activation. Int J Mol Sci (2022) 23(7):3750. doi: 10.3390/ijms23073750 35409108 PMC8998215

[49] Ma Y Ye Y Zhang J Ruan CC Gao PJ. Immune imbalance is associated with the development of preeclampsia. Med (Baltimore) (2019) 98(14):e15080. doi: 10.1097/MD.0000000000015080 PMC6455976 30946359

[50] Dela Justina V Gonçalves JS de Freitas RA Fonseca AD Volpato GT Tostes RC. Increased O-linked N-acetylglucosamine modification of NF-κB and augmented cytokine production in the placentas from hyperglycemic rats. Inflammation (2017) 40(5):1773–81. doi: 10.1007/s10753-017-0620-7 28688099

[51] Haider S Knöfler M. Human tumour necrosis factor: physiological and pathological roles in placenta and endometrium. Placenta (2009) 30(2):111–23. doi: 10.1016/j.placenta.2008.10.012 PMC2974215 19027157

[52] Otun HA Lash GE Innes BA Bulmer JN Naruse K Hannon T. Effect of tumour necrosis factor-α in combination with interferon-γ on first trimester extravillous trophoblast invasion. J Reprod Immunol (2011) 88(1):1–11. doi: 10.1016/j.jri.2010.10.003 21112094

[53] Chistiakov DA Killingsworth MC Myasoedova VA Orekhov AN Bobryshev YV. CD68/macrosialin: not just a histochemical marker. Lab Invest (2017) 97(1):4–13. doi: 10.1038/labinvest.2016.116 27869795

[54] Kim EN Yoon BH Jeon EJ Lee JB Hong JS Lee JY. Placental deposition of C-reactive protein is a common feature of human pregnancy. Placenta (2015) 36(6):704–7. doi: 10.1016/j.placenta.2015.03.006 25817719

[55] Li C Zhou J Huang Z Pan X Leung W Chen L. The clinical value and variation of antithyroid antibodies during pregnancy. Dis Markers (2020) 2020:8871951. doi: 10.1155/2020/8871951 33144894 PMC7599418

[56] Peoc'h K Puy V Fournier T. Haem oxygenases play a pivotal role in placental physiology and pathology. Hum Reprod Update (2020) 26(5):634–49. doi: 10.1093/humupd/dmaa014 32347305

[57] Siwetz M Blaschitz A El-Heliebi A Hiden U Desoye G Huppertz B. TNF-α alters the inflammatory secretion profile of human first trimester placenta. Lab Invest (2016) 96(4):428–38. doi: 10.1038/labinvest.2015.159 26752743

[58] Yu N Wu JL Xiao J Fan L Chen SH Li W. HIF-1α regulates angiogenesis via Notch1/STAT3/ETBR pathway in trophoblastic cells. Cell Cycle (2019) 18(24):3502–12. doi: 10.1080/15384101.2019.1689481 PMC6927703 31724455

[59] Bastida-Ruiz D Yart L Wuillemin C Ribaux P Morris N Epiney M. The fine-tuning of endoplasmic reticulum stress response and autophagy activation during trophoblast syncytialization. Cell Death Dis (2019) 10(9):651. doi: 10.1038/s41419-019-1905-6 31501418 PMC6733854

[60] Fradet S Pierredon S Ribaux P Epiney M Shin Ya K Irion O. Involvement of membrane GRP78 in trophoblastic cell fusion. PloS One (2012) 7(8):e40596. doi: 10.1371/journal.pone.0040596 22912664 PMC3415408

[61] Bastida-Ruiz D Wuillemin C Pederencino A Yaron M Martinez de Tejada B Pizzo SV. Activated α2-macroglobulin binding to cell surface GRP78 induces trophoblastic cell fusion. Sci Rep (2020) 10(1):9666. doi: 10.1038/s41598-020-66554-0 32541810 PMC7295802

[62] Khong TY Mooney EE Ariel I Balmus NC Boyd TK Brundler MA. Sampling and definitions of placental lesions: amsterdam placental workshop group consensus statement. Arch Pathol Lab Med (2016) 140(7):698–713. doi: 10.5858/arpa.2015-0225-CC 27223167

[63] Di Girolamo R Liberati M Silvi C D'Antonio F. Levothyroxine supplementation in euthyroid pregnant women with positive autoantibodies: A systematic review and meta-analysis. Front Endocrinol (Lausanne) (2022) 13:759064. doi: 10.3389/fendo.2022.759064 35250850 PMC8892207

[64] Habibi N Labrinidis A Leemaqz SY Jankovic-Karasoulos T McCullough D Grieger JA. Effect of selenium and iodine on oxidative stress in the first trimester human placenta explants. Nutrients (2021) 13(3):800. doi: 10.3390/nu13030800 33671070 PMC7997475

[65] Wang JQ Hu YB Liang CM Xia X Li ZJ Gao H. Aluminum and magnesium status during pregnancy and placenta oxidative stress and inflammatory mRNA expression: China Ma'anshan birth cohort study. Environ Geochem Health (2020) 42(11):3887–98. doi: 10.1007/s10653-020-00619-x 32621275

[66] Wang JQ Hu YB Gao H Sheng J Huang K Zhang YW. Sex-specific difference in placental inflammatory transcriptional biomarkers of maternal phthalate exposure: a prospective cohort study. J Expo Sci Environ Epidemiol (2020) 30(5):835–44. doi: 10.1038/s41370-020-0200-z 32015430

