# Supplementary figures and images for: Association of maternal thyroid peroxidase antibody during pregnancy with placental morphology and inflammatory and oxidative stress responses
Source: Front Endocrinol (Lausanne). 2023 Sep 22;14:1182049. doi: 10.3389/fendo.2023.1182049 (PMC10556745; doi:10.3389/fendo.2023.1182049)

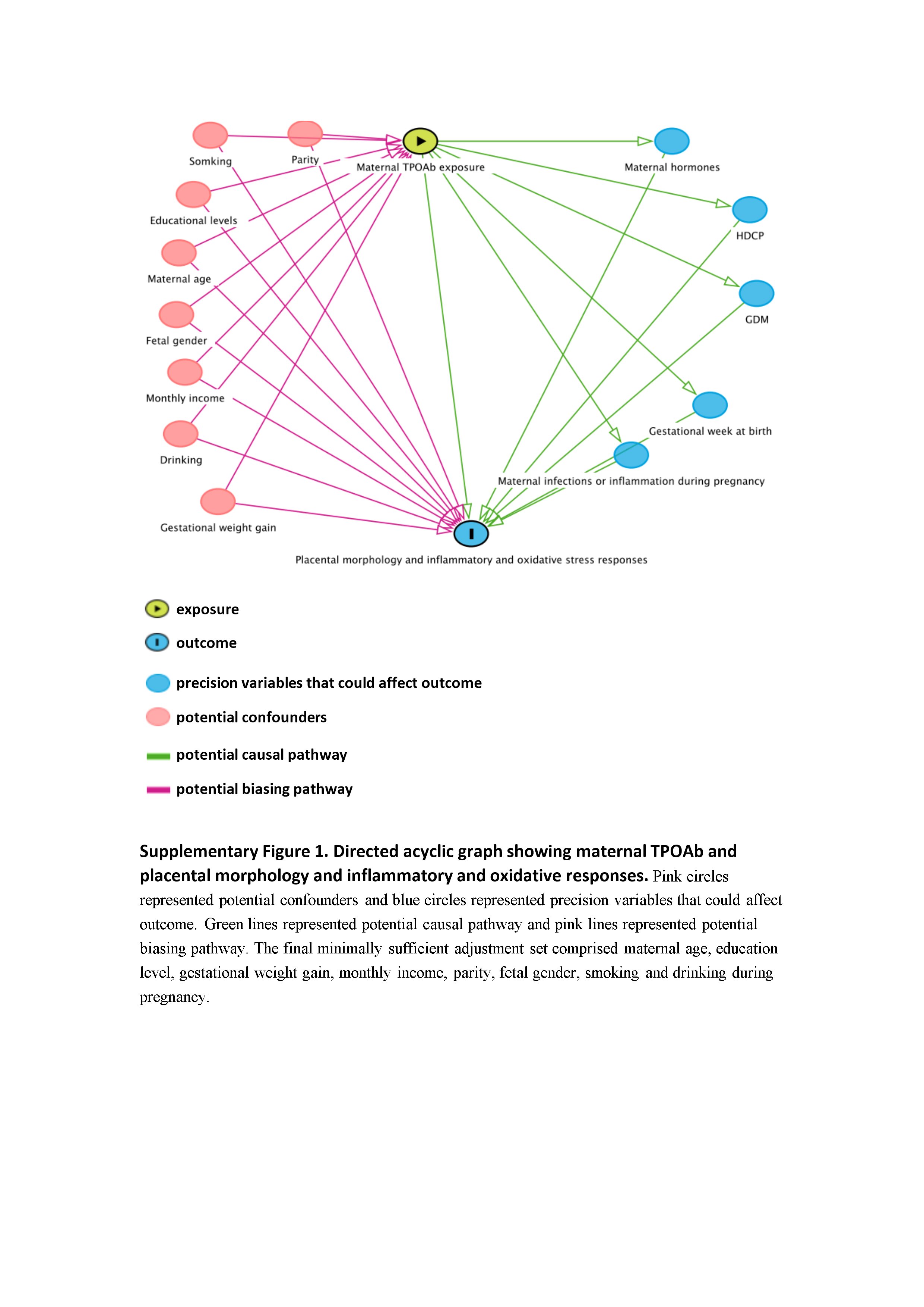

Supplement: Supplementary file 1 [file Image_1.jpeg]
