# Supplementary material for: Association of maternal thyroid peroxidase antibody during pregnancy with placental morphology and inflammatory and oxidative stress responses
Source: Front Endocrinol (Lausanne). 2023 Sep 22;14:1182049. doi: 10.3389/fendo.2023.1182049 (PMC10556745; doi:10.3389/fendo.2023.1182049)
Supplement: Supplementary file 2 [file Table_1.docx]

Supplementary Table 1 Sensitivity analysis: Association (β and 95% confidence intervals) of maternal TPOAb exposure (IU/mL) and placental morphological indicators (n=2274).

|  | **Models** | **Placental**  **Length** | **Placental**  **Width** | **Placental Thickness** | **Placental**  **Area** | **Placental**  **Volume** | **Placental**  **Weight** | **Disc**  **Eccentricity** |
| --- | --- | --- | --- | --- | --- | --- | --- | --- |
| TPOAb_a | 1 | -0.002  (-0.009,0.005) | **0.009**  **(0.001,0.017)*** | **-0.025**  **(-0.038,-0.011)**** | 0.007  (-0.006,0.020) | -0.018  (-0.037,0.001) | -0.018  (-0.037,0.001) | **-0.011**  **(-0.018,-0.004)**** |
|  | 2 | **-0.006**  **(-0.011,-0.001)*** | 0.001  (-0.005,0.006) | **-0.010**  **(-0.020,-0.001)*** | -0.006  (-0.015,0.003) | **-0.016**  **(-0.029,-0.002)*** | **-0.016**  **(-0.029,-0.002)*** | **-0.007**  **(-0.012,-0.002)*** |
|  | 3 | **-0.006**  **(-0.011,-0.001)*** | 0.001  (-0.004,0.007) | **-0.010**  **(-0.020,-0.001)*** | -0.005  (-0.014,0.004) | **-0.016**  **(-0.029,-0.002)*** | **-0.016**  **(-0.029,-0.002)*** | **-0.007**  **(-0.013,-0.002)**** |
|  | 4 | **-0.006**  **(-0.011,-0.001)*** | 0.001  (-0.005,0.006) | **-0.010**  **(-0.020,-0.001)*** | -0.005  (-0.015,0.004) | **-0.016**  **(-0.029,-0.002)*** | **-0.016**  **(-0.029,-0.002)*** | **-0.007**  **(-0.012,-0.002)*** |
|  | 5 | **-0.006**  **(-0.011,-0.001)*** | 0.002  (-0.004,0.007) | -0.009  (-0.019,0.001) | -0.004  (-0.013,0.005) | -0.013  (-0.026,0.001) | -0.013  (-0.026,0.001) | **-0.007**  **(-0.013,-0.002)*** |
| TPOAb_b | 1 | 0.001  (-0.008,0.011) | -0.009  (-0.019,0.001) | **0.034**  **(0.016,0.052)**** | -0.008  (-0.024,0.009) | **0.026**  **(0.001,0.051)*** | **0.026**  **(0.001,0.051)*** | 0.010  (0.0004,0.020)* |
|  | 2 | **-0.007**  **(-0.013,-0.001)*** | **-0.007**  **(-0.014,-0.0001)*** | 0.010  (-0.002,0.022) | **-0.014**  **(-0.025,-0.003)*** | -0.004  (-0.021,0.013) | -0.004  (-0.021,0.013) | -0.0001  (-0.007,0.007) |
|  | 3 | **-0.007**  **(-0.013,-0.001)*** | -0.006  (-0.013,0.0004) | 0.009  (-0.003,0.021) | **-0.013**  **(-0.025,-0.002)*** | -0.004  (-0.021,0.013) | -0.004  (-0.021,0.013) | -0.001  (-0.007,0.006) |
|  | 4 | **-0.007**  **(-0.013,-0.002)*** | **-0.007**  **(-0.014,-0.00001)*** | 0.010  (-0.002,0.022) | **-0.014**  **(-0.025,-0.002)*** | -0.004  (-0.021,0.013) | -0.004  (-0.021,0.013) | -0.0001  (-0.007,0.007) |
|  | 5 | **-0.006**  **(-0.013,-0.001)*** | **-0.006**  **(-0.013,0.001)*** | 0.010  (-0.001,0.022) | **-0.013**  **(-0.024,-0.001)*** | -0.002  (-0.019,0.015) | -0.002  (-0.019,0.015) | -0.0003  (-0.007,0.006) |
| TPOAb_c | 1 | -0.008  (-0.018,0.002) | -0.007  (-0.018,0.004) | -0.005  (-0.024,0.014) | -0.015  (-0.033,0.003) | -0.020  (-0.047,0.007) | -0.020  (-0.047,0.007) | -0.001  (-0.012,0.009) |
|  | 2 | **-0.010**  **(-0.018,-0.003)**** | -0.007  (-0.014,0.001) | -0.002  (-0.015,0.012) | **-0.017**  **(-0.030,-0.005)**** | **-0.019**  **(-0.038,-0.00004)*** | **-0.019**  **(-0.038,-0.00004)*** | -0.004  (-0.011,0.004) |
|  | 3 | **-0.011**  **(-0.018,-0.004)**** | -0.006  (-0.014,0.001) | -0.002  (-0.016,0.011) | **-0.017**  **(-0.030,-0.004)**** | **-0.019**  **(-0.038,-0.0003)*** | **-0.019**  **(-0.038,-0.0003)*** | -0.004  (-0.012,0.003) |
|  | 4 | **-0.011**  **(-0.018,-0.003)**** | -0.007  (-0.015,0.001) | -0.002  (-0.015,0.012) | **-0.017**  **(-0.030,-0.005)**** | **-0.019**  **(-0.038,-0.0002)*** | **-0.019**  **(-0.038,-0.0002)*** | -0.004  (-0.011,0.004) |
|  | 5 | **-0.010**  **(-0.017,-0.003)**** | -0.006  (-0.014,0.001) | -0.001  (-0.014,0.012) | **-0.016**  **(-0.029,-0.004)**** | -0.017  (-0.036,0.001) | -0.017  (-0.036,0.001) | -0.004  (-0.011,0.004) |
| a: First Trimester; b: Second Trimester; c: Third Trimester.  Model 1: adjusted for TPOAb concentrations in the other two trimesters, and the concentrations of TSH and FT4 in this trimester based on main analysis.  Model 2: adjusted for GDM based on main analysis.  Model 3: adjusted for HDCP based on main analysis.  Model 4: adjusted for maternal infection or inflammation during pregnancy based on main analysis.  Model 5: adjusted for gestational age at birth based on main analysis.  *: *P* < 0.05. **: *P* < 0.01. | | | | | | | | |

Supplementary Table 2 Sensitivity analysis: Associations (β and 95% confidence intervals) between TPOAb positivity and placental morphological indicators in repeated measure analysis (n=2274).

|  | **Models** | **Placental Length** | **Placental Width** | **Placental Thickness** | **Placental Area** | **Placental Volume** | **Placental Weight** | **Disc Eccentricity** |
| --- | --- | --- | --- | --- | --- | --- | --- | --- |
| TPOAb+ | Main | **-0.012**  **(-0.021,**  **-0.003)*** | **-0.011**  **(-0.021,**  **-0.002)*** | 0.012  (-0.005,  0.029) | **-0.023**  **(-0.039,**  **-0.007)**** | -0.011  (-0.036,  0.013) | -0.011  (-0.036,  0.013) | -0.001  (-0.010,  0.009) |
|  | 1 | **-0.011**  **(-0.020,**  **-0.002)*** | **-0.011**  **(-0.021,**  **-0.001)*** | 0.014  (-0.004,  0.031) | **-0.022**  **(-0.038,**  **-0.005)**** | -0.008  (-0.033,  0.016) | -0.008  (-0.033,  0.016) | 0.001  (-0.009,  0.010) |
|  | 2 | **-0.012**  **(-0.021,**  **-0.003)*** | **-0.011**  **(-0.021,**  **-0.002)*** | 0.012  (-0.005,  0.029) | **-0.023**  **(-0.039,**  **-0.007)**** | -0.011  (-0.036,  0.013) | -0.011  (-0.036,  0.013) | -0.001  (-0.010,  0.009) |
|  | 3 | **-0.012**  **(-0.024,**  **-0.001)*** | **-0.010**  **(-0.020,**  **-0.0002)*** | 0.011  (-0.006,  0.028) | **-0.022**  **(-0.039,**  **-0.006)**** | -0.011  (-0.036,  0.013) | -0.011  (-0.036,  0.013) | -0.002  (-0.012,  0.007) |
|  | 4 | **-0.012**  **(-0.021,**  **-0.003)*** | **-0.011**  **(-0.021,**  **-0.001)*** | 0.012  (-0.005,  0.029) | **-0.023**  **(-0.039,**  **-0.007)**** | -0.011  (-0.036,  0.013) | -0.011  (-0.036,  0.013) | -0.001  (-0.010,  0.009) |
|  | 5 | **-0.011**  **(-0.020,**  **-0.002)*** | -0.009  (-0.019,  0.0004) | 0.014  (-0.003,  0.032) | **-0.020**  **(-0.036,**  **-0.004)**** | -0.006  (-0.030,  0.019) | -0.006  (-0.030,  0.019) | -0.001  (-0.011,  0.008) |
| Control groups: TPOAb negativity.  Model main: djusted for maternal age, maternal education years, gestational weight gain, monthly income, parity, smoking, drinking and fetal gender.  Model 1: adjusted for the concentrations of TSH and FT_4_ based on main analysis.  Model 2: adjusted for GDM based on main analysis.  Model 3: adjusted for HDCP based on main analysis.  Model 4: adjusted for maternal infection or inflammation during pregnancy based on main analysis.  Model 5: adjusted for gestational age at birth based on main analysis.  *: *P* < 0.05. **: *P* < 0.01. | | | | | | | | |

Supplementary Table 3 Sensitivity analysis: Association (β and 95% confidence intervals) between TPOAb levels (IU/mL) and placental morphological indicators in repeated measure analysis (n=2274).

|  | **Models** | **Placental Length** | **Placental Width** | **Placental Thickness** | **Placental Area** | **Placental Volume** | **Placental Weight** | **Disc Eccentricity** |
| --- | --- | --- | --- | --- | --- | --- | --- | --- |
| TPOAb | Main | **-0.007**  **(-0.010,**  **-0.004)**** | -0.003  (-0.007,  0.0004) | -0.002  (-0.008,  0.004) | **-0.010**  **(-0.016,**  **-0.004)**** | **-0.012**  **(-0.021,**  **-0.003)**** | **-0.012**  **(-0.021,**  **-0.003)**** | **-0.004**  **(-0.007,**  **-0.0004)*** |
|  | 1 | **-0.006**  **(-0.009,**  **-0.003)**** | -0.003  (-0.007,  0.001) | -0.001  (-0.008,  0.005) | **-0.009**  **(-0.015,**  **-0.003)**** | **-0.010**  **(-0.020,**  **-0.001)**** | **-0.010**  **(-0.020,**  **-0.001)**** | -0.003  (-0.007,  0.0004) |
|  | 2 | **-0.007**  **(-0.011,**  **-0.004)**** | -0.003  (-0.007,  0.0004) | -0.002  (-0.009,  0.004) | **-0.010**  **(-0.016,**  **-0.004)**** | **-0.012**  **(-0.022,**  **-0.003)**** | **-0.012**  **(-0.022,**  **-0.003)**** | **-0.004**  **(-0.007,**  **-0.0003)*** |
|  | 3 | **-0.007**  **(-0.011,**  **-0.004)**** | -0.004  (-0.006,  0.001) | -0.002  (-0.009,  0.004) | **-0.010**  **(-0.016,**  **-0.004)**** | **-0.012**  **(-0.022,**  **-0.003)**** | **-0.012**  **(-0.022,**  **-0.003)**** | **-0.004**  **(-0.008,**  **-0.001)*** |
|  | 4 | **-0.007**  **(-0.010,**  **-0.004)**** | -0.003  (-0.007,  0.0004) | -0.002  (-0.009,  0.004) | **-0.010**  **(-0.016,**  **-0.004)**** | **-0.012**  **(-0.021,**  **-0.003)**** | **-0.012**  **(-0.021,**  **-0.003)**** | **-0.004**  **(-0.007,**  **-0.0004)*** |
|  | 5 | **-0.007**  **(-0.010,**  **-0.003)**** | -0.002  (-0.006,  0.0001) | -0.001  (-0.008,  0.005) | **-0.009**  **(-0.015,**  **-0.003)**** | **-0.010**  **(-0.019,**  **-0.001)**** | **-0.010**  **(-0.019,**  **-0.001)**** | **-0.004**  **(-0.008,**  **-0.001)*** |
| Model main: djusted for maternal age, maternal education years, gestational weight gain, monthly income, parity, smoking, drinking and fetal gender.  Model 1: adjusted for the concentrations of TSH and FT4 based on main analysis.  Model 2: adjusted for GDM based on main analysis.  Model 3: adjusted for HDCP based on main analysis.  Model 4: adjusted for maternal infection or inflammation during pregnancy based on main analysis.  Model 5: adjusted for gestational age at birth based on main analysis.  *: *P* < 0.05. **: *P* < 0.01. | | | | | | | | |

Supplementary Table 4 Sensitivity analysis: Association (β and 95% confidence intervals) of maternal TPOAb exposure (IU/mL) and placental inflammatory and oxidative stress cytokines (n=2122).

|  | **Models** | **IL-1β** | **IL-6** | **TNF-α** | **IFN-γ** | **CRP** | **CD68** | **MCP-1** | **IL-4** | **IL-10** | **HO-1** | **HIF-1α** | **GRP78** |
| --- | --- | --- | --- | --- | --- | --- | --- | --- | --- | --- | --- | --- | --- |
| TPOAb_a | 1 | **0.176**  **(0.077,**  **0.275)**** | **0.313**  **(0.221,**  **0.405)**** | **0.230**  **(0.126,**  **0.334)**** | -0.0002  (-0.121,  0.121) | **0.330**  **(0.175,**  **0.485)**** | **0.562**  **(0.432,**  **0.692)**** | **0.361**  **(0.269,**  **0.454)**** | -0.057  (-0.161,  0.048) | **0.250**  **(0.142,**  **0.359)**** | **0.329**  **(0.231,**  **0.427)**** | **0.373**  **(0.276,**  **0.470)**** | **0.572**  **(0.438,**  **0.705)**** |
|  | 2 | 0.067  (-0.005,  0.138) | **0.193**  **(0.127,**  **0.259)**** | **0.162**  **(0.087,**  **0.237)**** | 0.035  (-0.052,  0.122) | **0.165**  **(0.053,**  **0.277)**** | **0.369**  **(0.275,**  **0.463)**** | **0.198**  **(0.131,**  **0.265)**** | 0.003  (-0.072,  0.078) | **0.142**  **(0.063,**  **0.220)**** | **0.168**  **(0.098,**  **0.239)**** | **0.175**  **(0.104,**  **0.245)**** | **0.285**  **(0.187,**  **0.382)**** |
|  | 3 | 0.066  (-0.005,  0.138) | **0.193**  **(0.127,**  **0.260)**** | **0.160**  **(0.085,**  **0.235)**** | 0.032  (-0.055,  0.120) | **0.167**  **(0.055,**  **0.279)**** | **0.368**  **(0.274,**  **0.462)**** | **0.197**  **(0.130,**  **0.264)**** | 0.003  (-0.073,  0.078) | **0.142**  **(0.064,**  **0.221)**** | **0.168**  **(0.097,**  **0.239)**** | **0.175**  **(0.104,**  **0.246)**** | **0.285**  **(0.187,**  **0.382)**** |
|  | 4 | 0.066  (-0.005,  0.137) | **0.193**  **(0.127,**  **0.259)**** | **0.161**  **(0.086,**  **0.236)**** | 0.034  (-0.053,  0.121) | **0.165**  **(0.053,**  **0.277)**** | **0.370**  **(0.276,**  **0.464)**** | **0.198**  **(0.131,**  **0.265)**** | 0.003  (-0.072,  0.078) | **0.142**  **(0.064,**  **0.220)**** | **0.168**  **(0.097,**  **0.238）**** | **0.175**  **(0.105,**  **0.246)**** | **0.286**  **(0.188,**  **0.383)**** |
|  | 5 | 0.066  (-0.006,  0.137) | **0.191**  **(0.125,**  **0.258)**** | **0.166**  **(0.091,**  **0.241)**** | 0.035  (-0.052,  0.123) | **0.168**  **(0.056,**  **0.280)**** | **0.370**  **(0.276,**  **0.464)**** | **0.201**  **(0.134,**  **0.268)**** | -0.002  (-0.078,  0.074) | **0.147**  **(0.068,**  **0.225)**** | **0.172**  **(0.101,**  **0.243)**** | **0.177**  **(0.106,**  **0.248)**** | **0.290**  **(0.192,**  **0.387)**** |
| TPOAb_b | 1 | **-0136**  **(-0.265,**  **-0.008)*** | -0.100  (-0.219,  0.020) | -0.078  (-0.214,  0.057) | 0.100  (-0.057,  0.258) | **-0.228**  **(-0.431,**  **-0.026)*** | **-0.222**  **(-0.391,**  **-0.052)*** | **-0.228**  **(-0.349,**  **0.107)**** | 0.055  (-0.082,  0.191) | **-0.212**  **(-0.353,**  **0.070)**** | **-0.240**  **(-0.368,**  **0.113)**** | **-0.332**  **(-0.458,**  **0.205)**** | **-0.451**  **(-0.625,**  **0.276)**** |
|  | 2 | -0.051  (-0.139,  0.036) | 0.049  (-0.033,  0.131) | 0.058  (-0.034,  0.150) | 0.080  (-0.026,  0.187) | -0.033  (-0.170,  0.105) | 0.106  (-0.011,  0.223) | -0.002  (-0.085,  0.081) | 0.047  (-0.045,  0.139) | -0.009  (-0.105,  0.087) | -0.032  (-0.119,  0.055) | -0.074  (-0.161,  0.013) | -0.078  (-0.198,  0.042) |
|  | 3 | -0.051  (-0.139,  0.036) | 0.049  (-0.033,  0.131) | 0.057  (-0.035,  0.149) | 0.079  (-0.028,  0.185) | -0.032  (-0.170,  0.105) | 0.103  (-0.014,  0.220) | -0.004  (-0.087,  0.079) | 0.048  (-0.044,  0.141) | -0.010  (-0.107,  0.086) | -0.033  (-0.120,  0.055) | -0.075  (-0.163,  0.012) | -0.080  (-0.200,  0.040) |
|  | 4 | -0.051  (-0.139,  0.036) | 0.049  (-0.033,  0.131) | 0.058  (-0.034,  0.150) | 0.080  (-0.027,  0.187) | -0.033  (-0.170,  0.105) | 0.106  (-0.011,  0.223) | -0.002  (-0.085,  0.081) | 0.047  (-0.045,  0.139) | -0.009  (-0.105,  0.087) | -0.032  (-0.119,  0.055) | -0.074  (-0.161,  0.013) | -0.078  (-0.198,  0.043) |
|  | 5 | -0.052  (-0.139,  0.036) | 0.049  (-0.033,  0.131) | 0.058  (-0.034,  0.150) | 0.080  (-0.027,  0.187) | -0.033  (-0.170,  0.105) | 0.106  (-0.011,  0.223) | -0.002  (-0.085,  0.081) | 0.047  (-0.045,  0.139) | -0.009  (-0.105,  0.087) | -0.032  (-0.119,  0.055) | -0.074  (-0.161,  0.013) | -0.078  (-0.198,  0.043) |
| TPOAb_c | 1 | -0.110  (-0.251,  0.030) | **-0.181**  **(-0.312,**  **-0.051)**** | -0.107  (-0.255,  0.040) | -0.037  (-0.210,  0.135) | -0.117  (-0.338,  0.105) | **-0.249**  **(-0.434,**  **-0.063)**** | -0.109  (-0.241,  0.023) | 0.057  (-0.092,  0.207) | 0.014  (-0.141  0.169) | -0.087  (-0.226,  0.052) | -0.086  (-0.225,  0.052) | -0.174  (-0.365,  0.016) |
|  | 2 | -0.051  (-0.149,  0.048) | 0.020  (-0.072,  0.112) | 0.048  (-0.055,  0.152) | 0.042  (-0.078,  0.163) | -0.002  (-0.157,  0.152) | 0.097  (-0.035,  0.228) | 0.031  (-0.063,  0.124) | 0.051  (-0.052,  0.155) | 0.063  (-0.045,  0.171) | 0.011  (-0.086,  0.109) | -0.006  (-0.104,  0.091) | -0.006  (-0.141,  0.130) |
|  | 3 | -0.051  (-0.149,  0.048) | 0.020  (-0.072,  0.112) | 0.048  (-0.055,  0.152) | 0.041  (-0.079,  0.161) | -0.004  (-0.158,  0.151) | 0.094  (-0.038,  0.225) | 0.029  (-0.065,  0.122) | 0.053  (-0.050,  0.157) | 0.061  (-0.048,  0.169) | 0.010  (-0.088,  0.108) | -0.009  (-0.107,  0.089) | -0.009  (-0.144,  0.127) |
|  | 4 | -0.050  (-0.148,  0.048) | 0.020  (-0.072,  0.112) | 0.049  (-0.054,  0.153) | 0.043  (-0.077,  0.163) | -0.003  (-0.157,  0.152) | 0.096  (-0.035,  0.228) | 0.030  (-0.063,  0.124) | 0.052  (-0.052,  0.155) | 0.062  (-0.046,  0.170) | 0.012  (-0.086,  0.109) | -0.007  (-0.105,  0.091) | -0.006  (-0.142,  0.129) |
|  | 5 | -0.051  (-0.149,  0.047) | 0.019  (-0.073,  0.111) | 0.051  (-0.053,  0.154) | 0.044  (-0.077,  0.164) | -0.004  (-0.159,  0.151) | 0.096  (-0.036,  0.227) | 0.031  (-0.062,  0.124) | 0.051  (-0.053,  0.155) | 0.063  (-0.045,  0.171) | 0.012  (-0.086,  0.110) | -0.007  (-0.105,  0.091) | -0.006  (-0.141,  0.130) |
| a: First Trimester; b: Second Trimester; c: Third Trimester.  Model 1: adjusted for TPOAb concentrations in the other two trimesters, and the concentrations of TSH and FT4 in this trimester based on main analysis.  Model 2: adjusted for GDM based on main analysis.  Model 3: adjusted for HDCP based on main analysis.  Model 4: adjusted for maternal infection or inflammation during pregnancy based on main analysis.  Model 5: adjusted for gestational age at birth based on main analysis.  *: *P* < 0.05. **: *P* < 0.01. | | | | | | | | | | | | | |

Supplementary Table 5 Sensitivity analysis: Associations (β and 95% confidence intervals) between TPOAb positivity and placental inflammatory and oxidative stress cytokines in repeated measure analysis (n=2122).

|  | Models | IL-1β | IL-6 | TNF-α | IFN-γ | CRP | CD68 | MCP-1 | IL-4 | IL-10 | HO-1 | HIF-1α | GRP78 |
| --- | --- | --- | --- | --- | --- | --- | --- | --- | --- | --- | --- | --- | --- |
| TPOAb+ | Main | -0.099  (-0.227,  0.030) | 0.119  (-0.001,  0.240) | **0.137**  **(0.002,**  **0.272)*** | 0.005  (-0.152,  0.162) | 0.137  (-0.065,  0.339) | **0.270**  **(0.098,**  **0.442)**** | **0.144**  **(0.022,**  **0.266)*** | 0.022  (-0.114,  0.158) | 0.076  (-0.065,  0.217) | 0.107  (-0.021,  0.235) | 0.027  (-0.101,  0.155) | 0.089  (-0.088,  0.266) |
|  | 1 | -0.113  (-0.243,  0.017) | 0.105  (-0.016,  0.227) | 0.105  (-0.032,  0.242) | 0.011  (-0.148,  0.169) | 0.136  (-0.069,  0.340) | **0.229**  **(0.055,**  **0.402)*** | **0.126**  **(0.003,**  **0.249)*** | 0.025  (-0.113,  0.162) | 0.067  (-0.076,  0.210) | 0.092  (-0.038,  0.221) | -0.001  (-0.131,  0.128) | 0.040  (-0.138,  0.212) |
|  | 2 | -0.099  (-0.227,  0.030) | 0.119  (-0.001,  0.240) | **0.137**  **(0.002,**  **0.273)*** | 0.006  (-0.151,  0.163) | 0.136  (-0.066,  0.338) | **0.270**  **(0.098,**  **0.441)**** | **0.144**  **(0.022,**  **0.265)*** | 0.022  (-0.113,  0.158) | 0.075  (-0.066,  0.216) | 0.106  (-0.022,  0.234) | 0.026  (-0.102,  0.154) | 0.088  (-0.089,  0.264) |
|  | 3 | -0.100  (-0.228,  0.029) | 0.121  (-0.0002,  0.241) | 0.134  (-0.002,  0.269) | -0.001  (-0.158,  0.156) | 0.138  (-0.065,  0.340) | **0.262**  **(0.090,**  **0.434)**** | **0.139**  **(0.017,**  **0.261)*** | 0.026  (-0.110,  0.161) | 0.072  (-0.069,  0.214) | 0.104  (-0.024,  0.232) | 0.023  (-0.105,  0.151) | 0.082  (-0.095,  0.259) |
|  | 4 | -0.099  (-0.228,  0.029) | 0.119  (-0.001,  0.240) | **0.137**  **(0.001,**  **0.272)*** | 0.005  (-0.152,  0.162) | 0.136  (-0.065,  0.338) | **0.270**  **(0.098,**  **0.442)**** | **0.144**  **(0.022,**  **0.266)*** | 0.022  (-0.113,  0.158) | 0.076  (-0.065,  0.217) | 0.107  (-0.021,  0.234) | 0.027  (-0.101,  0.155) | 0.089  (-0.088,  0.266) |
|  | 5 | -0.102  (-0.230,  0.027) | 0.114  (-0.007,  0.234) | **0.145**  **(0.010,**  **0.280)*** | 0.007  (-0.149,  0.165) | 0.137  (-0.065,  0.340) | **0.267**  **(0.095,**  **0.439)**** | **0.148**  **(0.026,**  **0.269)*** | 0.014  (-0.122,  0.150) | 0.082  (-0.060,  0.223) | 0.111  (-0.017,  0.240) | 0.028  (-0.100,  0.156) | 0.093  (-0.084,  0.270) |
| Control groups: TPOAb negative.  Model main: djusted for maternal age, maternal education years, gestational weight gain, monthly income, parity, smoking, drinking and fetal gender.  Model 1: adjusted for the concentrations of TSH and FT4 based on main analysis.  Model 2: adjusted for GDM based on main analysis.  Model 3: adjusted for HDCP based on main analysis.  Model 4: adjusted for maternal infection or inflammation during pregnancy based on main analysis.  Model 5: adjusted for gestational age at birth based on main analysis.  *: *P* < 0.05. **: *P* < 0.01. | | | | | | | | | | | | | |

Supplementary Table 6 Sensitivity analysis: Associations (β and 95% confidence intervals) between TPOAb levels (IU/mL) and placental inflammatory and oxidative stress cytokines in repeated measure analysis (n=2122).

|  | **Models** | **IL-1β** | **IL-6** | **TNF-α** | **IFN-γ** | **CRP** | **CD68** | **MCP-1** | **IL-4** | **IL-10** | **HO-1** | **HIF-1α** | **GRP78** |
| --- | --- | --- | --- | --- | --- | --- | --- | --- | --- | --- | --- | --- | --- |
| TPOAb | Main | 0.003  (-0.045,  0.050) | **0.103**  **(0.059,**  **0.147)**** | **0.099**  **(0.049,**  **0.148)**** | 0.048  (-0.009,  0.106) | 0.063  (-0.012,  0.137) | **0.215**  **(0.152,**  **0.278)**** | **0.093**  **(0.048,**  **0.138)**** | 0.026  (-0.023,  0.076) | **0.074**  **(0.022,**  **0.126)**** | **0.067**  **(0.020,**  **0.114)**** | **0.054**  **(0.007,**  **0.101)*** | **0.101**  **(0.036,**  **0.166)**** |
|  | 1 | -0.106  (-0.059,  0.038) | **0.097**  **(0.052,**  **0.142)**** | **0.083**  **(0.032,**  **0.272)**** | 0.046  (-0.013,  0.105) | 0.060  (-0.016,  0.135) | **0.195**  **(0.130,**  **0.259)**** | **0.084**  **(0.038,**  **0.129)**** | 0.027  (-0.023,  0.078) | **0.074**  **(0.020,**  **0.127)**** | **0.062**  **(0.014,**  **0.110)*** | 0.039  (-0.009,  0.087) | **0.078**  **(0.011,**  **0.143)*** |
|  | 2 | 0.003  (-0.045,  0.050) | **0.103**  **(0.059,**  **0.147)**** | **0.099**  **(0.049,**  **0.149)**** | 0.048  (-0.009,  0.106) | 0.062  (-0.012,  0.136) | **0.215**  **(0.152,**  **0.278)**** | **0.093**  **(0.048,**  **0.138)**** | 0.027  (-0.023,  0.077) | **0.074**  **(0.022,**  **0.126)**** | **0.067**  **(0.020,**  **0.114)**** | **0.053**  **(0.006,**  **0.100)*** | **0.101**  **(0.036,**  **0.166)**** |
|  | 3 | 0.002  (-0.045,  0.050) | **0.104**  **(0.059,**  **0.148)**** | **0.098**  **(0.048,**  **0.148)**** | 0.047  (-0.011,  0.104) | 0.063  (-0.012,  0.137) | **0.213**  **(0.150,**  **0.276)**** | **0.092**  **(0.047,**  **0.136)**** | 0.028  (-0.023,  0.077) | **0.073**  **(0.021,**  **0.125)**** | **0.066**  **(0.019,**  **0.114)**** | **0.053**  **(0.006,**  **0.100)*** | **0.100**  **(0.034,**  **0.164)**** |
|  | 4 | 0.002  (-0.045,  0.050) | **0.103**  **(0.059,**  **0.148)**** | **0.099**  **(0.049,**  **0.148)**** | 0.048  (-0.010,  0.106) | 0.062  (-0.012,  0.136) | **0.215**  **(0.152,**  **0.278)**** | **0.093**  **(0.048,**  **0.138)**** | 0.027  (-0.023,  0.077) | **0.074**  **(0.022,**  **0.126)**** | **0.067**  **(0.020,**  **0.114)**** | **0.054**  **(0.006,**  **0.100)*** | **0.101**  **(0.036,**  **0.166)**** |
|  | 5 | 0.002  (-0.046,  0.049) | **0.101**  **(0.057,**  **0.146)**** | **0.102**  **(0.052,**  **0.151)**** | 0.049  (-0.009,  0.107) | 0.062  (-0.012,  0.137) | **0.214**  **(0.151,**  **0.277)**** | **0.094**  **(0.050,**  **0.139)**** | 0.024  (-0.026,  0.074) | **0.076**  **(0.024,**  **0.128)**** | **0.069**  **(0.022,**  **0.116)**** | **0.054**  **(0.007,**  **0.101)*** | **0.103**  **(0.038,**  **0.168)**** |
| Model main: djusted for maternal age, maternal education years, gestational weight gain, monthly income, parity, smoking, drinking and fetal gender.  Model 1: adjusted for the concentrations of TSH and FT4 based on main analysis.  Model 2: adjusted for GDM based on main analysis.  Model 3: adjusted for HDCP based on main analysis.  Model 4: adjusted for maternal infection or inflammation during pregnancy based on main analysis.  Model 5: adjusted for gestational age at birth based on main analysis.  *: *P* < 0.05. **: *P* < 0.01. | | | | | | | | | | | | | |
